# A Randomized Controlled Clinical Trial Investigating the Weaning Process From Mechanical Ventilation in Elderly Patients With Dementia

**DOI:** 10.1111/crj.13808

**Published:** 2024-07-16

**Authors:** Jian‐Feng Liang, Zhi‐Yong Li, Hai‐Shan Dong, Chang Xu, Chao‐Qun Yin

**Affiliations:** ^1^ Department of Intensive Care Unit Beijing Geriatric Hospital Beijing China

**Keywords:** dementia, elderly patients, intensive care unit, mechanical ventilation, weaning

## Abstract

**Background:**

Limited data is available regarding the weaning techniques employed for mechanical ventilation (MV) in elderly patients with dementia in China.

**Objective:**

The primary objective of this study is to investigate diverse weaning methods in relation to the prognostic outcomes of elderly patients with dementia undergoing MV in the intensive care unit (ICU). Specifically, we seek to compare the prognosis, likelihood of successful withdrawal from MV, and the length of stay (LOS) in the ICU.

**Methods:**

The study was conducted as a randomized controlled trial, encompassing a group of 169 elderly patients aged ≥ 65 years with dementia who underwent MV. Three distinct weaning methods were used for MV cessation, namely, the tapering parameter, spontaneous breathing trial (SBT), and SmartCare (Dräger, Germany).

**Results:**

In the tapering parameter group, the LOS in the ICU was notably prolonged compared to both the SBT and SmartCare groups. However, no statistically significant differences were observed among the groups with respect to demographic characteristics, such as age and sex, as well as factors including the rationale for ICU admission, cause of MV, MV mode, oxygenation index, hemoglobin levels, albumin levels, ejection fraction, sedation and analgesia practices, tracheotomy, duration of MV, successful extubation, successful weaning, incidences of ventilator‐associated pneumonia, and overall prognosis.

**Conclusions:**

Both the SBT and SmartCare withdrawal methods demonstrated a reduction in the duration of MV and LOS in the ICU when compared to the tapering parameter method.

**Trial Registration:**

Chinese Clinical Trial Registry: ChiCTR1900028449

## Graphical abstract

This study is aimed at investigating the weaning techniques for mechanical ventilation in elderly patients with dementia in China. Three methods were compared: tapering parameter, spontaneous breathing trial (SBT), and SmartCare. Results showed that SBT and SmartCare led to shorter ICU stays compared to the tapering parameter method. No significant differences were found in demographic characteristics or prognostic outcomes among the groups.

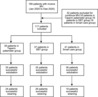

**Abbreviations**
APACHE II: Acute Physiology and Chronic Health Evaluation II
ARDS: acute respiratory distress syndrome
CVP: central venous pressure
ICU: intensive care unit
IQR: interquartile range
LOS: length of stay
MV: mechanical ventilation
NPPV: noninvasive positive pressure mechanical ventilation
OR: odds ratios
SBT: spontaneous breathing trial (defined as a T‐piece trial or a low‐level pressure support ≤ 8 cmH_2_O)
SD: standard deviation
VAP: ventilator‐associated pneumonia

## 1 Introduction

The use of mechanical ventilation (MV) plays a pivotal role in the critical care management of patients. Consequently, scholars have long been preoccupied with examining the strategy employed in the application of MV. However, in recent years, there has been a shift in scholarly focus toward studying MV, transitioning from the selection of ventilation modes to exploring weaning methods. Many scholars now recognize that the ultimate goal of MV lies in the weaning process, a concern applicable to all patients undergoing MV. The initial investigations into MV weaning date back to the 1990s. In 1994, Esteban et al. [1] conducted a study examining the weaning methods utilized for 290 patients across 47 intensive care units (ICUs) in Spain, revealing that distinct withdrawal methods led to varying weaning durations. Subsequently, in 1995, Esteban et al. [2] proposed the use of the spontaneous breathing trial (SBT) as a method to reduce MV duration in patients facing challenges with weaning, enhance the success rate of weaning, and decrease reintubation rates. In 1997 [3], two different SBT methods were compared, with an analysis of the distinctive characteristics of patients undergoing SBT. In 1999, a study [4] indicated that a 30‐min SBT achieved comparable results to a 2‐h SBT in terms of ventilator efficacy, advocating for the clinical application of the former as a substitute for the latter. In subsequent years, the quest for the optimal weaning method became a focal point in the field of MV. Researchers explored the causes of MV, encompassing conditions such as acute respiratory distress syndrome (ARDS), chronic obstructive pulmonary disease, asthma, or postoperative respiratory dysfunction. However, a notable gap exists in the research concerning elderly patients with dementia who experience impaired brain function. Consequently, the most advantageous weaning method for this specific demographic remains unclear.

Globally, the prevalence of dementia is estimated at 47 million individuals, and it is projected to rise to 131 million by the year 2050. Despite this increasing burden, the effectiveness of available treatments remains constrained [5]. Dementia, being incurable, is currently managed through symptomatic interventions [6]. As the aging demographic in China grows, there has been a gradual rise in the number of patients with dementia. Moreover, the incidence of pulmonary infections in patients with dementia necessitating MV has concurrently increased. Weaning poses a challenge for elderly patients with impaired brain function. To address this challenge, the ICU at Beijing Geriatric Hospital has been employing empirical and SBT withdrawal methods for such patients. Recent advancements in high‐precision signal detection technology, using microprocessors (computers and sensors), have given rise to a novel ventilator type—the intelligent ventilation system (SmartCare). This technology is progressively finding application in clinical settings. However, the optimal ventilator type for elderly patients with dementia remains an area requiring exploration. This study is aimed at determining the most suitable weaning method for patients with senile dementia by comparing empirical withdrawal, autonomous breathing test withdrawal, and intelligent withdrawal methods. The objective is to enhance the success rate of weaning, diminish ICU duration, and enhance patient prognosis.

## 2 Materials and Methods

### 2.1 Study Design and Patients

The randomized controlled trial was conducted at Beijing Geriatric Hospital (Beijing, China), a tertiary hospital managed by the Beijing Hospital Administration Center. The management of the 15‐bed ICU was overseen by full‐time ICU directors, and the staff included 6 physicians and 25 nurses.

The inclusion criteria for this study were as follows: (1) admission to the ICU of Beijing Geriatric Hospital between January 2020 and December 2020; (2) diagnosis of dementia upon admission to the ICU; (3) age ≥ 65 years; (4) receiving invasive MV with the establishment of an artificial airway (endotracheal intubation) via the mouth, nose, or tracheotomy; and (5) receiving MV for more than 48 h. The exclusion criteria were as follows: (1) ventilator dependency (at the end of the study, MV continued for more than 3 months, and the withdrawal process could not be initiated), (2) discontinuation or abandonment of treatment, and (3) death (refers to patients who were unable to proceed to the weaning procedure and were not able to undergo the final analysis, and these patients were already randomized to each group at the time of randomization).

The types of dementia comprised of Alzheimer's disease, frontotemporal lobar dementia, dementia with Lewy bodies, Parkinson's disease dementia, and vascular dementia.

The inclusion criteria clarified that patients need to meet 48 h of invasive MV after admission to the ICU. This study is a single‐center study, and the study is independent and has no conflicts of interest. Randomization was carried out using a random table.

The tapering parameter group method is operated by the doctor in charge; according to the patient's vital signs and blood gas analysis results, gradually reduce the patient's ventilator support parameters, including pressure, volume, respiratory rate, and oxygen concentration, until the weaning conditions acceptable to the doctor in charge are reached, and the endotracheal intubation is removed.

The SBT group method is operated according to the standard of SBT, using a low‐level positive pressure support method; that is, the ventilator is adjusted to PS mode, which meets the specific parameter standards of SBT, including pressure, positive end‐expiratory pressure and inspired oxygen concentration, and if the patient has successful SBT, the endotracheal intubation is removed.

The SmartCare group is a software function that comes with the ventilator; according to the monitored patient's respiratory rate, tidal volume, and end‐tidal carbon dioxide level, the ventilator uses voluntary feedback to gradually reduce the support pressure level until the target level is reached, and the alarm notifies the doctor to wean.

Indications for invasive MV in our hospital encompass the following: (1) deterioration of clinical condition after active treatment of underlying diseases; (2) disturbance of consciousness; (3) severe respiratory abnormalities, such as a respiratory rate > 35–40 bpm or < 6–8 bpm, abnormal respiratory rhythm, or weak/absent spontaneous breathing; and (4) severe ventilation and oxygenation disorders indicated by blood gas analysis (PaO_2_ < 50 mm Hg, progressively elevated PaCO_2_, and progressive acidosis).

**FIGURE 1:**
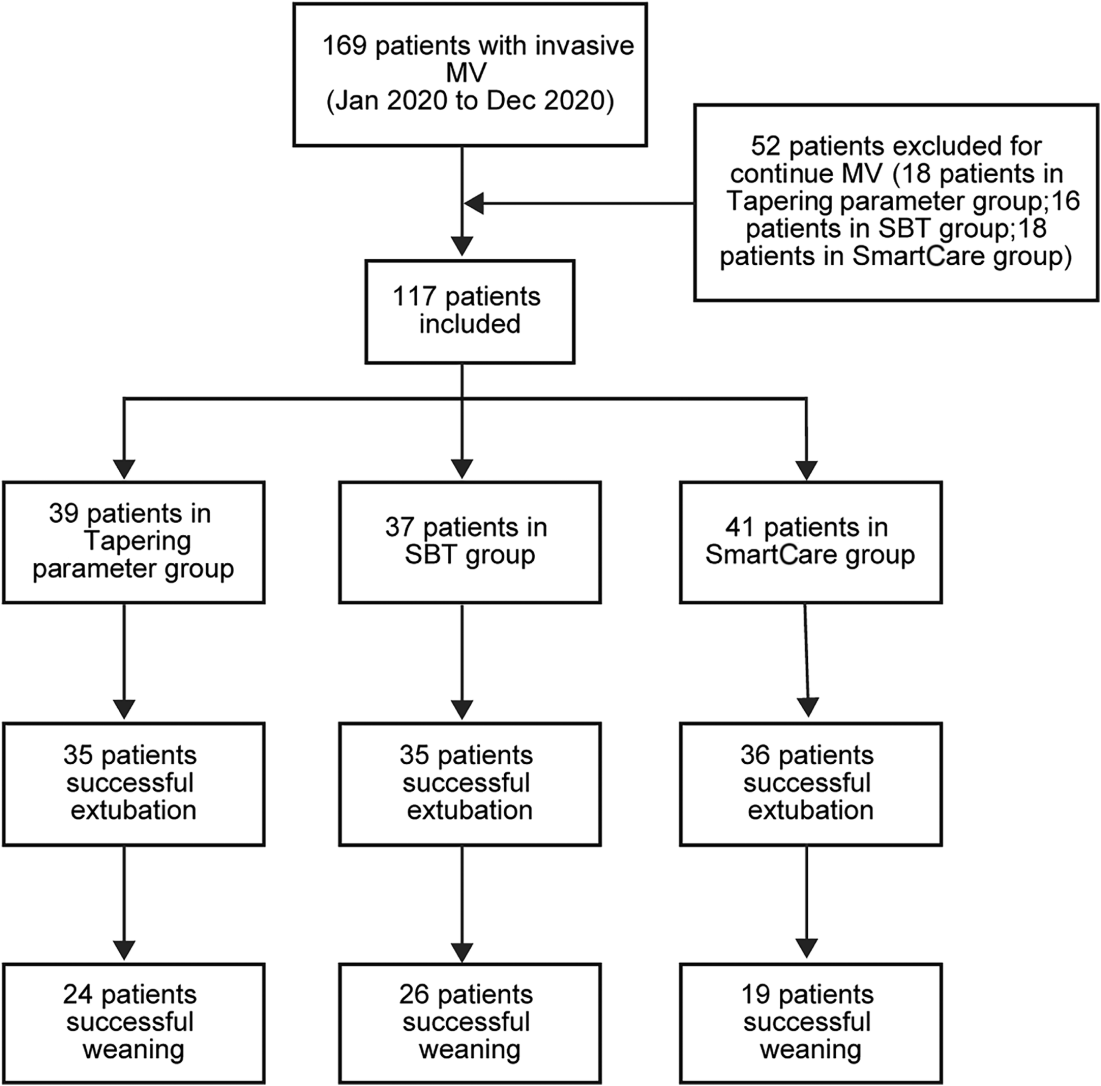
Flow chart depicting enrollment of the study participants.

This study was conducted in compliance with the Declaration of Helsinki (2000) established by the World Medical Association. All medical interventions were executed with the informed consent of the patients or their family members. Approval for this study was obtained from the Institutional Ethical Committee of Beijing Geriatric Hospital under the reference number 2018BJLNYY‐Ethical‐2018‐001. The trial was preregistered before patient enrollment, with Jian‐Feng Liang as the principal investigator and the registration date being December 22, 2019. The patient enrollment process is illustrated in Figure 1.

### 2.2 Collection of Clinical Data

The medical records were used to extract the following data: age, sex, Acute Physiology and Chronic Health Evaluation II (APACHE II) score [7], reasons for ICU admission, reasons for MV, MV mode, oxygenation index in MV, hemoglobin levels in MV, albumin levels in MV, ejection fraction in MV, sedation, analgesia, tracheotomy, duration of MV, hemoglobin levels during extubation, albumin levels during extubation, ejection fraction during extubation, outcomes of extubation attempts (successful or unsuccessful), outcomes of weaning attempts (successful or unsuccessful), instances of withdrawal machine failure (necessitating MV, including reintubation and noninvasive positive pressure ventilation [NPPV], within 48 h) [2], reasons for the failure to wean, occurrences of ventilator‐associated pneumonia (VAP) defined in accordance with established criteria [8], LOS in the ICU, and prognosis.

### 2.3 Statistical Analysis

The analysis of data was performed using SPSS 17 (SPSS Inc., Illinois, United States). Descriptive statistics included presenting frequency (percentages) for categorical variables, while mean and standard deviation or median and interquartile ranges (IQRs) were used for continuous variables. Proportions were compared using chi‐squared or exact Fisher tests, and continuous variables were subjected to comparison using the Mann–Whitney *U* test or analysis of variance. Odds ratios (ORs) and corresponding 95% confidence intervals (95% CIs) were calculated. Statistical significance was determined at a *p* value < 0.05.

### 2.4 Patient and Public Involvement

No members of the public or patients themselves were engaged in any way with the planning, execution, reporting, or dissemination of our study.

### 2.5 The Results of the Study

The main result is that, despite no statistically significant difference, the actual duration of MV in the tapering parameter group was significantly longer than that in the SBT and SmartCare groups. The secondary result is that the LOS in the ICU was much greater in the tapering parameter group compared to the SBT and SmartCare groups.

## 3 Results

### 3.1 Patient Characteristics

During the period from January 2020 to December 2020, 169 elderly patients with dementia underwent invasive MV in the ICU of our hospital. The enrollment duration spanned 12 months (January 1, 2020, to December 31, 2020), and the study extended over 15 months (January 1, 2020, to March 31, 2021). Among these patients, 52 were excluded from the study due to ventilator dependency (*n* = 2, who never initiated a weaning process), discontinuation of treatment (*n* = 3), or death (*n* = 47). Consequently, 117 patients were included in the study (Figure 1).

The baseline characteristics of the study participants are presented in Table 1. The 117 patients (87 male patients, 74.4%) had a median age of 82 (IQR, 74.5–86) years and an average APACHE II score of 24.3 ± 7.6. The tapering parameter group comprised of 39 patients (32 male patients, 82.1%) with a median age of 83 (IQR, 78–87) years and an average APACHE II score of 21.9 ± 8.0. The SBT group included 37 cases (25 male patients, 67.6%) with a median age of 79 (IQR, 73–85.5) years and an average APACHE II score of 24.8 ± 6.9. The SmartCare group consisted of 41 cases (30 male patients, 73.2%) with a median age of 82 (IQR, 65.8–86) years and an average APACHE II score of 26.2 ± 7.3. No significant differences were observed in age and the proportion of male patients among the three groups (Table 1). However, in comparison with the tapering parameter group (means gradually reducing the parameters of the ventilator until MV is removed), both the SBT and SmartCare groups exhibited a moderately higher APACHE II score (*p* = 0.03). The statistical significance of the APACHE II score among the three groups was considered related to the limited number of enrolled cases. No statistically significant differences were identified among the three groups concerning the reason for ICU admission (*p* = 0.678), reason for MV (*p* = 0.890), MV mode (*p* = 0.199), oxygenation index (*p* = 0.424), hemoglobin (*p* = 0.390), albumin (*p* = 0.155), ejection fraction (*p* = 0.618), sedation (*p* = 0.397), analgesia (*p* = 0.626), tracheotomy (*p* = 0.547), and duration of MV (*p* = 0.273) (Table 1).

**TABLE 1:** Clinical characteristics of the study participants.

	Total (*n* = 117)	Tapering parameter group (*n* = 39)	SBT group (*n* = 37)	Smart care group (*n* = 41)	*p*
Age (year), median (IQR)	82.0 (74.5–86.0)	83.0 (78.0–87.0)	79.0 (73.0–85.5)	82.0 (68.5–86.0)	0.14
Male sex	87 (74.4%)	32 (82.1%)	25 (67.6%)	30 (73.2%)	0.344
APACHE II score, mean ± SD	24.3 ± 7.6	21.9 ± 8.0	24.8 ± 6.9	26.2 ± 7.3	0.03
Reason for admission to the ICU					0.678
Respiratory disorder	73 (62.4%)	25 (64.1%)	25 (67.6%)	23 (56.1%)	
Surgery (i.e., postoperative)	21 (17.9%)	7 (17.9%)	7 (18.9%)	7 (17.1%)	
Other	23 (19.7%)	7 (17.9%)	5 (13.5%)	11 (26.8%)	
Reasons for MV					0.890
Respiratory failure	80 (68.4%)	27 (69.2%)	25 (67.6%)	28 (68.3%)	
Surgery (i.e., postoperative)	21 (17.9%)	7 (17.9%)	8 (21.6%)	6 (14.6%)	
Other	16 (13.7%)	5 (12.8%)	4 (10.8%)	7 (17.1%)	
MV modes					0.199
Pressure support	79 (67.5%)	27 (69.2%)	21 (56.8%)	31 (75.6%)	
Volume support	38 (32.5%)	12 (30.8%)	16 (43.2%)	10 (24.4%)	
Oxygenation index in MV	217.6 ± 91.3	210.1 ± 91.1	234.0 ± 97.3	210.0 ± 86.1	0.424
Hemoglobin in MV	107.1 ± 27.4	107.5 ± 22.8	111.5 ± 29.3	102.9 ± 29.7	0.390
Albumin in MV	29.9 ± 5.6	30.1 ± 5.5	31.1 ± 5.0	28.6 ± 6.2	0.155
Ejection fraction in MV	59.2 ± 5.6	59.8 ± 5.4	58.6 ± 6.4	59.1 ± 5.0	0.618
Sedation	44 (37.6%)	18 (46.2%)	12 (32.4%)	14 (34.1%)	0.397
Analgesia	27 (23.1%)	7 (17.9%)	9 (24.3%)	11 (26.8%)	0.626
Tracheotomy	19 (16.2%)	7 (17.9%)	4 (10.8%)	8 (19.5%)	0.547
Duration of MV (h), median (IQR)	182.0 (93.5–315.5)	232.0 (92.0–404.0)	141.0 (98.0–292.0)	169.0 (86.5–427.5)	0.273

### 3.2 Clinical Characteristics of the Extubation Participants

Upon the removal of tracheal intubation, there were no statistically significant differences observed among the three groups with regard to hemoglobin levels (*p* = 0.381) and ejection fraction (*p* = 0.469). When compared to the SmartCare group, both the tapering parameter and SBT groups exhibited a moderately higher albumin level (*p* = 0.029). The *p* value of albumin between the three groups was statistically significant, which was also considered related to the limited number of enrolled cases (Table 2).

**TABLE 2:** Clinical characteristics of the extubation participants.

	Total (*n* = 106)	Tapering parameter group (*n* = 35)	SBT group (*n* = 35)	Smart care group (*n* = 36)	*p*
Hemoglobin during extubation	95.1 ± 21.1	95.1 ± 16.2	98.6 ± 24.2	91.6 ± 21.9	0.381
Albumin during extubation	30.9 ± 4.6	31.0 ± 4.7	32.3 ± 4.7	29.4 ± 4.2	0.029
Ejection fraction during extubation, median (IQR)	60.5 (56.8–63.0)	62.0 (58.0–64.0)	61.0 (55.0–63.0)	60.0 (56.0–63.8)	0.469

### 3.3 Clinical Characteristics of the Outcomes of Patients Undergoing MV

There was no statistically significant difference between the three groups in terms of the likelihood of a successful extubation (*p* = 0.576) or weaning (*p* = 0.135). (Table 3).

**TABLE 3:** Clinical characteristics of the outcomes of patients undergoing MV.

	Total (*n* = 117)	Tapering parameter group (*n* = 39)	SBT group (*n* = 37)	Smart care group (*n* = 41)	*p*
Successful extubation	106 (90.6%)	35 (89.7%)	35 (94.6%)	36 (87.8%)	0.576
Successful weaning	69 (65.1%)	24 (68.6%)	26 (74.3%)	19 (52.8%)	0.143

### 3.4 Reason for the Failure to Wean and Extubate

Causes of extubation failure (*p* = 0.990) and weaning failure (*p* = 0.945) were not significantly different among the three groups (Table 4).

**TABLE 4:** Reasons for the failure to wean and dispose.

	Total (*n* = 37)	Tapering parameter group (*n* = 11)	SBT group (*n* = 9)	Smart care group (*n* = 17)	*p*
Withdrawal machine failure^a^					0.990
Reintubation	13 (35.1%)	4 (36.4%)	3 (33.3%)	6 (35.3%)	
NPPV	24 (64.9%)	7 (63.6%)	6 (66.7%)	11 (64.7%)	
Reasons for the failure to wean					0.945
Respiratory failure	14 (37.8%)	5 (45.5%)	3 (33.3%)	6 (35.3%)	
Heart failure	9 (24.3%)	2 (18.2%)	2 (22.2%)	5 (29.4%)	
Inadequate airway protection capability or center driver disorder	14 (37.8%)	4 (36.4%)	4 (44.4%)	6 (35.3%)	

^a^ Withdrawal machine failure was defined as the need for MV (including reintubation and NPPV) within 48 h.

### 3.5 Complication and Prognosis

Between the three groups, there was no statistically significant difference in VAP incidence (*p* = 0.692) or prognosis (*p* = 0.342). However, LOS in the ICU was clearly distinct (*p* = 0.013). The LOS in the ICU was much greater in the tapering parameter group compared to the SBT and SmartCare groups (Table 5).

**TABLE 5:** Complication and prognosis.

	Total (*n* = 117)	Tapering parameter group (*n* = 39)	SBT group (*n* = 37)	Smart care group (*n* = 41)	*p*
Ventilator‐associated pneumonia	21 (17.9%)	8 (20.5%)	5 (13.5%)	8 (19.5%)	0.692
Length of stay in the intensive care unit (day)	21.9 ± 22.5	30.5 ± 32.7	17.2 ± 11.0	17.9 ± 15.5	0.013
Prognosis					0.342
Survivors	88 (75.2%)	28 (71.8%)	31 (83.8%)	29 (70.7%)	
Nonsurvivors	29 (24.8%)	11 (28.2%)	6 (16.2%)	12 (29.3%)	

## 4 Discussion

In the year 2020, we undertook a comparative analysis of the impact of three distinct withdrawal methods on the prognostic outcomes of elderly patients with senile dementia who were undergoing MV in the ICU at Beijing Geriatric Hospital. Out of the initially enrolled 169 patients, 52 were subsequently excluded—comprising 18 from the tapering parameter group, 16 from the SBT group, and 18 from the SmartCare group. Previous studies [9, 10] demonstrated that the pressure support ventilation (PSV) mode of SBT had a higher rate of successful extubation without an increased risk of reintubation when compared to the T‐piece mode of SBT. The PSV mode of SBT for withdrawal tests in the ICU was employed in the present study. A total of 117 patients passed daily screening and proceeded to the weaning process, with 39, 37, and 41 patients assigned to the tapering parameter, SBT, and SmartCare groups, respectively. As detailed in Table 1, the baseline characteristics of patients in the three groups were comparable in terms of age, sex, reason for ICU admission, reason for MV, MV mode, oxygenation index during MV, hemoglobin during MV, albumin during MV, ejection fraction during MV, sedation, analgesia, tracheotomy, and duration of MV. The APACHE II score, widely used for assessing the severity of patients who are critically ill and prognostic assessment in ICUs, was also included. A statistically significant difference (*p* = 0.03) in APACHE II scores was noted, likely attributed to the relatively low number of enrolled patients and the random nature of patient selection. Following the withdrawal procedure, tracheal intubation was successfully removed in 35, 35, and 36 patients in the tapering parameter, SBT, and SmartCare groups, respectively. While no difference was observed in hemoglobin and myocardial ejection fraction during extubation, a statistically significant difference was identified in albumin levels (*p* = 0.029), which was deemed to be associated with the small sample size. Following the removal of endotracheal intubation, 24, 26, and 19 patients in the tapering parameter, SBT, and SmartCare groups were successfully weaned from the ventilator, respectively. No statistically significant difference was detected in the rates of successful extubation and successful weaning among the three groups (Table 3). Eleven patients in the tapering parameter group, 9 in the SBT group, and 17 in the SmartCare group encountered difficulty in being successfully weaned from MV. Subsequently, they underwent reintubation or received noninvasive ventilator‐assisted treatment within 48 h due to various reasons after the removal of endotracheal intubation.

Table 4 lists the reasons for the failure to wean and subsequent extubation. No statistically significant differences were identified among the three patient groups concerning the three reasons for weaning failure, encompassing respiratory failure, heart failure, inadequate airway protection capability, or central driver disorder. Additionally, no statistically significant differences were observed between the three groups in terms of the use of endotracheal intubation or noninvasive ventilator assistance after withdrawal machine failure. One study [11] indicated that weaning‐induced pulmonary edema contributed to an increased rate of weaning failure. Other studies discovered that ΔPCO_2_ and central venous oxygen saturation during SBT were independent predictors of weaning outcomes [12]. Furthermore, an early elevation in central venous pressure (CVP) emerged as a promising indicator for identifying patients at high risk of weaning failure [13]. A systematic review and meta‐analysis indicated that brain natriuretic peptides could predict successful liberation from MV [14], and diastolic dysfunction was identified as a predictor of weaning failure [15]. Consequently, meticulous attention to relevant clinical indicators before the withdrawal process may offer a more accurate prediction of the withdrawal outcome.

As is evident in Table 5, there are no statistically significant difference in the incidence of VAP and the prognosis among the three groups. VAP is documented as the most prevalent infection in patients undergoing MV, with an incidence ranging from 9% to 27% [16]. VAP is associated with increased mortality and higher medical costs [17]. Various factors, such as trauma, prior surgery, ARDS, chronic obstructive pulmonary disease, upper airway colonization, duration of MV, and use of proton pump inhibitors (PPIs), are considered contributors to VAP progression [16]. Patients undergoing prolonged MV are at a heightened risk of VAP. Given the absence of a difference in MV duration among the three groups, no variance was observed in the incidence of VAP. There was no difference between the SBT and SmartCare groups regarding the LOS indicators in the ICU. However, the ICU LOS in the tapering parameter group was notably longer than that in the other groups. The potential explanation lies in the fact that, in the tapering parameter group, the attending physician needed to gradually reduce the level of ventilator support, relying to a greater extent on subjective factors. Delays in the weaning process occurred when the attending physician could not promptly adjust the set parameters or in situations where the physician was occupied or absent. In contrast, in the SBT and SmartCare groups, the attending physician initiated the weaning process based on predefined time nodes, facilitating a more streamlined weaning progression. These reasons were also reflected in the duration of MV, where, despite no statistically significant difference, the actual duration of MV in the tapering parameter group was significantly longer than that in the other two groups—232 h versus 141 and 169 h (Table 1). Two meta‐analyses concluded that compared to nonautomated weaning strategies, SmartCare reduced weaning time, time to successful extubation, and the length of ICU stay [17, 18]. However, conflicting results were reported in some studies, indicating that SmartCare did not significantly reduce the duration of MV [19], especially when using a 1:1 nurse‐to‐patient ratio [20], rendering it less advantageous in certain scenarios.

Over 20 years ago, studies revealed that the failure to wean patients undergoing MV was an independent risk factor associated with an increased risk of death in the ICU. Offline failure of MV in patients situated in the ICU was significantly linked to adverse outcomes. Successful withdrawal of patients in the ICU indicated an enhanced likelihood of a favorable prognosis. In a recent study [21], it was disclosed that ICU‐acquired weakness was identified in 38% of patients in a high‐risk population at the time of extubation and was independently associated with extubation failure in the ICU. Although this study did not assess weakness, some patients could not be weaned from MV due to this complication. Furthermore, the incidence of concurrent complications increased with prolonged MV time. Typical complications of MV encompassed elevated rates of tracheal injuries, nosocomial infections, increased work of breathing, hemodynamic suppression, ventilator‐induced diaphragmatic dysfunction, and VAP. These complications are anticipated to contribute to increased mortality. Therefore, any measure capable of reducing the duration of MV could potentially benefit patients. While this study was designed with these considerations in mind, regrettably, the obtained results indicate a lack of positive outcomes. Esteban et al. [22] observed that over time, the outcomes of patients undergoing MV enhanced, with mortality decreasing from 31% in 1998 to 28% in 2010. In this study, the mortality of such patients was 24.8%, aligning with the trend of a gradual decline in mortality.

Our study has certain limitations: (1) We did not collect and assess predictors that could contribute to withdrawal failure. (2) Being a single‐center study, there exists a potential for information and/or selection bias. (3) The study did not analyze similar characteristics of the excluded patients in the three groups, and the absence of these details does not help readers understand what exactly the population studied in this survey is. (4) The study did not record details of patients' prerandomization status, including ventilator mode, respiratory mechanical characteristics, and duration of ventilator use. (5) Details of preparation for weaning and extubation standards were not recorded and reported in this study. The range of underlying disease groups was limited, and the generalizability of the findings remains unknown. Furthermore, the analysis may have been impacted by unknown confounding factors affecting prognosis. To address these limitations, a multicenter, randomized controlled study with a larger sample size should be conducted in the future to clarify the factors influencing the prognosis of patients undergoing weaning methods in the ICU.

## 5 Conclusions

In summary, this study demonstrated that the SBT and SmartCare withdrawal methods resulted in a shorter duration of MV and a reduced LOS in the ICU. Consequently, this led to decreased medical costs, alleviated the economic burden on patients, and lowered the burden on medical insurance when compared with the tapering parameter method.

## Author Contributions

Conception and design of the research: Jian‐Feng Liang and Zhi‐Yong Li. Acquisition of data: Jian‐Feng Liang, Zhi‐Yong Li, Chang Xu, and Chao‐Qun Yin. Analysis and interpretation of the data: Jian‐Feng Liang. Statistical analysis: Jian‐Feng Liang, Hai‐Shan Dong, and Chang Xu. Writing of the manuscript: Jian‐Feng Liang and Hai‐Shan Dong. Critical revision of the manuscript for intellectual content: Jian‐Feng Liang, and Chao‐Qun Yin. All authors read and approved the final draft.

## Ethics Statement

This work has been carried out in accordance with the Declaration of Helsinki (2000) of the World Medical Association. The Institutional Ethical Committee of Beijing Geriatric Hospital approved this study (2018BJLNYY‐Ethical‐2018‐001). The trial was registered prior to patient enrollment (www.chictr.org.cn; principal investigator: Jian‐Feng Liang, date of registration: December 22, 2019).

## Consent

All medical interventions were performed with the informed consent of the patient or his/her family members.

## Conflicts of Interest

The authors declare no conflicts of interest.

## Data Availability

The data that support the findings of this study are available from the corresponding author upon reasonable request.
